# Positron Emission Tomography: A Tool for Identifying the Effects of Alcohol Dependence on the Brain

**Published:** 2003

**Authors:** Dean F. Wong, Atul Maini, Olivier G. Rousset, James Robert Brašić

**Affiliations:** Dean F. Wong, M.D., Ph.D., is a professor in the Division of Nuclear Medicine and the vice chair for research, administration, and training in the Russell H. Morgan Department of Radiology and Radiological Science, John Hopkins University School of Medicine, Baltimore, Maryland. He is also a professor of Environmental Health Sciences in the Division of Radiation Health Science, Department of Environmental Health Science, Johns Hopkins Bloomberg School of Public Health, Baltimore, Maryland. Atul Maini, M.D., Olivier G. Rousset, Ph.D., and James Robert Brašić M.D., M.P.H., all are postdoctoral fellows in the Division of Nuclear Medicine, Russell H. Morgan Department of Radiology and Radiological Science, Johns Hopkins University School of Medicine, Baltimore, Maryland. Dr. Brašić also is a clinical assistant attending in the Department of Psychiatry at Bellevue Hospital Center and an adjunct assistant professor of psychiatry at the New York University School of Medicine, New York, New York; an Essel Investigator with the Essel Foundation, Mamaroneck, New York, and with the National Alliance for Research on Schizophrenia and Depression (NARSAD) Great Neck, New York; and a member of the Medical Advisory Board of the Tourette Syndrome Association of Greater Washington, Silver Spring, Maryland

**Keywords:** positron emission tomography, chronic AODE (alcohol and other drug effects), neurobiological theory of AODU (alcohol and other drug use), AOD dependence, brain reward pathway, neuroimaging, excitatory neurotransmitters, hyperexcitability, GABA receptors, glutamate, dopamine, mesolimbic system, serotonin, endogenous opioids, glucose metabolism, cerebral blood flow, AODR (alcohol and other drug related) structural brain damage

## Abstract

To study alcohol’s effects on the structure and function of the brain in living human beings, researchers can use various imaging techniques. Positron emission tomography (PET) is a functional imaging approach used to study the metabolism and physiology of the brain. PET studies have found that both acute and chronic alcohol ingestion alter blood flow and metabolism in various brain regions, including the frontal lobes and cerebellum. Other analyses focusing on alcohol’s effects on brain chemical (i.e., neurotransmitter) systems have found that both acute and chronic alcohol consumption alter the activities of the inhibitory neurotransmitter gamma-aminobutyric acid (GABA) and the excitatory neurotransmitters glutamate, dopamine, and serotonin. These alterations may contribute to the reinforcing and rewarding effects of alcohol as well as to symptoms of alcohol withdrawal. Imaging studies also have demonstrated that some of alcohol’s adverse effects on brain function can be reversed by abstinence or alcoholism treatment interventions. In addition, imaging studies may help in the development of new medications for alcoholism treatment.

Alcohol exerts profound and harmful effects on the human nervous system. One way of determining how the brain is affected by alcohol consumption—particularly chronic excessive consumption that has led to alcohol dependence—is to look directly at the brain and its structures. Obviously, these examinations can be performed only during autopsies of deceased alcoholics. Investigations of the progression of alcohol-induced brain damage over time, its reversibility with abstinence, and the effectiveness of pharmacological and other interventions, however, require analyses in living subjects who can be studied repeatedly. Over the past few decades, various imaging techniques have been developed that allow researchers to study the structure and function of the brain both in healthy people and in people with alcoholism or other disorders. By allowing investigators to visualize alcohol’s actions on the brain in living human beings, these techniques are essential tools for documenting alcohol-induced damage as well as the effects of interventions for alcoholism.

This article focuses primarily on the contributions of one imaging technique—positron emission tomography (PET)—to the analysis of alcohol-related brain damage. Following a description of PET technology, the article explores how this approach has helped elucidate alcohol’s effects on the structures and functions of the brain, particularly its effects on various brain chemical (i.e., neurotransmitter) systems. Methodological considerations relevant to applying PET technology to studies of alcohol dependence also are discussed.

## PET and Other Neuroimaging Techniques

The various techniques to visually represent the nervous system that have been developed over the past few decades generally fall into two broad categories, structural and functional imaging approaches. Structural neuroimaging techniques, such as computerized tomography^1^ (CT), magnetic resonance imaging (MRI), and an MRI subtype known as diffusion tensor imaging (DTI), illustrate the anatomy of the nervous system. In alcohol research, these approaches are ideally suited for demonstrating anatomical changes that alcohol causes in the nervous system. In contrast, functional neuroimaging procedures—such as PET, functional MRI, magnetic resonance spectroscopy (MRS), and single photon emission computerized tomography (SPECT)— show the metabolic and physiologic processes of the nervous system in action. These imaging procedures are preferable for detecting alcohol-induced metabolic and physiologic alterations in the brain. Because each procedure has its strengths and weaknesses in the evaluation of people with alcoholism (Wong and Brašić 2001), clinicians and investigators must carefully consider the questions they want to address before deciding on the most appropriate approach.

**Figure 1:**
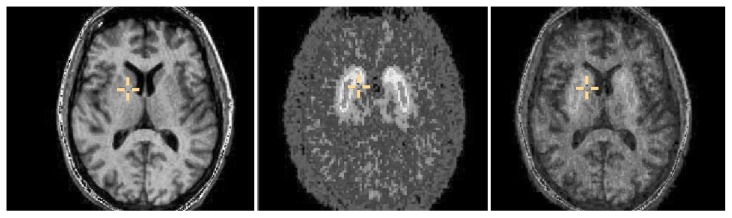
Pictures of the same level of the brain of a 40-year-old male alcoholic. **Left:** image obtained by magnetic resonance imaging (MRI). **Center:** view obtained by positron emission tomography (PET) after the administration of the agent [11C]raclopride, which binds to the dopamine receptor. **Right:** image resulting from the simultaneous combination of MRI and PET. Each picture shows the front of the brain at the top, the back of the brain at the bottom, the left side of the brain at the left, and the right side at the right of the picture. The cross in the images is located between two brain structures: the putamen, to the left of the cross, and the caudate, to the upper right. The MRI image clearly shows the anatomic structures. The PET image demonstrates that both the putamen and the caudate have high densities of dopamine receptors, as indicated by the yellow. However, the borders of these anatomical structures are blurred on the PET image, making them appear as a single structure. Superimposing the MRI and PET images yields an image that facilitates the identification of the distinct borders of anatomical structures such as the putamen and the caudate.

Structural and functional neuroimaging techniques may be combined for certain research questions. For example, consecutive structural and functional neuroimaging analyses can be used to determine the exact anatomic location of alcohol’s physiological and metabolic effects on the nervous system, and the results can be superimposed to obtain the most accurate estimates (Wong and Brašić 2001). An example of this procedure is the concomitant acquisition of both MRI (a structural technique) and PET (a functional technique) images on a person with alcoholism. The MRI and PET images then are realigned to obtain a composite image that has the benefits of the detailed structural information of MRI and the functional information from PET (see figure 1) (Wong and Brašić 2001).^2^

**Figure 2:**
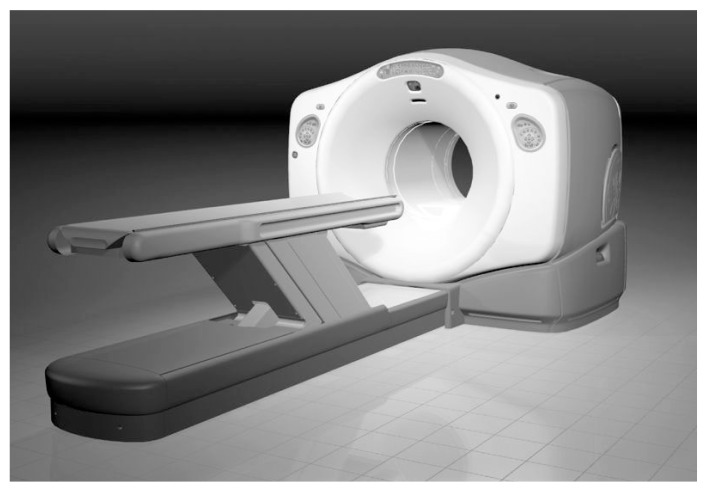
This state-of-the-art scanner is used to obtain both PET and CT images. SOURCE: Photograph provided courtesy of Dr. Alexander Y. Tokman, General Electric (GE) Medical Systems, Milwaukee, Wisconsin.

PET makes it possible to visualize the physiology of living human beings by tracking radioactive compounds (i.e., radiotracers) that are of potential biological importance in the body (Wong and Brašić 2001). A radiotracer is produced in the laboratory by attaching a radioactive atom or molecule to a compound of interest. It then is usually injected into the patient’s bloodstream, from which it can be taken up into the brain. This uptake of the radiotracer and its subsequent distribution within the brain can be measured over time to obtain information about the physiological process being studied. The amount of radiotracer administered is so small that it does not disturb the conditions in the living organism.^3^ As a result, one can get direct information on the process being studied by tracking the radioactive molecule using a measuring device called a PET scanner (see figure 2). In addition, one can obtain quantitative information about the biological processes as they occur in the living organism by processing the data with sophisticated computer software, which also can generate three-dimensional images of the structures where the radiotracer is found. (For more information on the technical details of PET, see the textbox, p. 166.)

**The Molecular Basis of Positron Emission Tomography (PET)**

Positrons and electrons are some of the tiny particles that make up atoms. As the name implies, positrons carry a positive electrical charge whereas electrons carry a negative electrical charge. Positrons are contained within the nucleus of each atom and can be released from atoms during the decay of unstable, radioactive atoms or molecules. The positrons can then be detected by scanners with sensitive cameras.

Radioactive decay is the basis of PET technology. The radioactive compounds required for PET (also called radiotracers) are generated in a cyclotron—a sophisticated machine to damage the nuclei of chemicals. Directly after their synthesis, the PET radiotracers already begin to decay and release positrons in the process. (Because the radiotracers used for PET generally decay very rapidly, PET is an extremely expensive procedure available only at selected facilities with or near cyclotrons.) Small amounts of the radiotracer are injected into the subject’s bloodstream, which distributes the tracer to the tissues, and the subject is placed in the PET scanner. During the radioactive decay of the radiotracer, the released positrons collide with electrons, resulting in the production of two particles of light (i.e., photons). Sensors within the PET scanner detect the photons, and attached computers with sophisticated software can use this information to identify the position of the original positrons. With the help of computed tomography obtained immediately before the PET (see figure 1), the computer can then generate three-dimensional images of the source of the photons (Wong and Brašić 2001). The computer also counts the collisions between positrons and electrons at each site in the brain, and these counts are proportional to the amount of radiotracer present at that site. For example, one can generate radiotracers that specifically bind to receptors for the neurotransmitters dopamine or serotonin. These radiotracers will bind to the receptors, with higher concentrations of the radiotracers accumulating in those brain regions that contain higher concentrations of the respective receptors. With this approach, investigators and clinicians can estimate the density and the distribution of particular neurotransmitter receptors in the living human brain. Currently available PET cameras can theoretically distinguish structures that are only 2 mm apart (Wong and Brašić 2001).

—Dean F. Wong, Atul Maini, Olivier G. Rousset, and James Robert Brašić

ReferenceWongDFBrašićJRIn vivo imaging of neurotransmitter systems in neuropsychiatryClinical Neuroscience Research135452001

To conduct functional brain imaging using PET, investigators need radiotracers that can cross the blood–brain barrier,^4^ distribute proportionally with the blood flow through the brain (i.e., regional cerebral blood flow [rCBF]), and remain in the brain long enough to permit PET imaging. PET tracers typically are identical or similar in structure (i.e., are analogs) to a naturally occurring molecule that acts specifically in the particular brain area, except that the radiotracers contain a radioactive atom. For example, the commonly used clinical radiotracer [^18^F]fluorodeoxyglucose (FDG) is an analog of the ordinary sugar, glucose, which serves as the source of energy in active brain cells. A tracer commonly used for research purposes is a radioactive antagonist of the neurotransmitter dopamine. This tracer can interact with proteins called dopamine receptors that are located on many nerve cells (neurons) and mediate dopamine’s actions on the cells (for more information on neurotransmitters and their actions, see the next section), but the antagonist’s effect is the opposite from that of dopamine. By measuring the levels of the radioactive dopamine antagonist in various brain regions, one can estimate how many dopamine receptors are present in those regions. For example, neurons in certain brain areas (e.g., the basal ganglia) carry particularly high numbers of dopamine receptors and are therefore especially likely to be governed by dopamine’s actions.

The radioactive atoms most commonly used in PET for studying the effect of alcohol on the brain are radioactive fluorine (^18^F), carbon (^11^C), and oxygen (^15^O). Of these, ^11^C, and ^15^O have relatively short half-lives of 20 minutes and 2 minutes, respectively. This means that after those times, only half of the original radioactivity remains in the radiotracers. As a result, PET radiotracers that incorporate ^11^C and ^15^O must be produced at the same site where the PET study is conducted to avoid losing most of the radioactivity before the patient is injected with the radiotracer. Radiotracers can be produced only by machines called cyclotrons, which are extremely expensive, bulky, and require radioactive shielding. Therefore, few facilities can afford to conduct PET analyses using ^11^C and ^15^O. In contrast, ^18^F has a relatively long half-life of 109 minutes, which together with the possibility of rapid regional transfer of ^18^F, permits the performance of FDG PET scans in many facilities without cyclotrons.

## Using PET to Determine Alcohol’s Effects on Brain Structure and Function

### Alcohol’s Acute Effects on the Brain

Both acute and chronic alcohol consumption can alter brain function—for example, changing blood flow through various brain regions and metabolic activities of those regions. PET and other neuroimaging approaches have detected such alterations, as follows:

- PET analyses following alcohol consumption in social drinkers showed reduced blood flow to the cerebellum, a region at the base of the brain that controls voluntary movements and coordination (Volkow et al. 1988). These findings may explain the muscular incoordination resulting from the consumption of alcohol.
- Acute alcohol ingestion reduces the metabolic activity of the brain. The pattern of this reduced activity suggests that alcohol increases nerve signal transmission through the inhibitory neurotransmitter gamma-aminobutyric acid (GABA) (Wang et al. 2003) (see the table). This effect is more pronounced in men than in women.

### Effects of Chronic Alcohol Consumption

Chronic alcohol consumption affects the brain both directly through its effects on brain cells and their functions and indirectly by causing nutritional deficiencies, liver disease, and disturbances of the hormonal and immune systems. Head trauma sustained during inebriation may also damage the brain. One approach commonly used to study the effects of long-term excessive alcohol consumption is to conduct autopsies of deceased alcoholics. Autopsy studies have demonstrated that people with a history of chronic alcohol consumption have smaller brains than age- and gender-matched nonalcoholics. Other autopsy studies have focused on alcoholics with Wernicke’s encephalopathy, a severe brain disease resulting from a deficiency of the vitamin thiamine that often is associated with chronic excessive alcohol consumption. These studies have shown marked reductions in the number of neurons in the outer layer of the upper surface of the front of the brain (i.e., the superior frontal cortex), particularly in patients with liver cirrhosis (Dodd et al. 1996). Additional autopsy studies of alcoholics with Wernicke’s encephalopathy have detected reduced numbers of neurons in the cerebellum (Baker et al. 1999).

**Table:** Neurotransmitters and Their Possible Roles in Alcohol Dependence

Neurotransmitter	Action in Health*	Action in Alcoholism	Reference
Acetylcholine	Conveys excitatory signals from one neuron to another	Chronic alcohol ingestion depresses the activity of ACh	Nevo and Hamon 1995
Adrenocorticotropic hormone (ACTH)	Conveys signals from the pituitary gland to the adrenal gland	Unknown	Terenius 1996
Beta–endorphin	Conveys signals from the pituitary gland to the adrenal gland	Unknown	Terenius 1996
Gamma-aminobutyric acid (GABA)	Conveys inhibitory signals from one neuron to another	Acute alcohol ingestion facilitates GABA’s inhibitory effect	Nevo and Hamon 1995; Wang et al. 2003
		Chronic alcohol ingestion reduces GABA’s inhibitory effect	Basavarajappa and Hungund 2002; Nevo and Hamon 1995
Bombesin	Conveys excitatory signals from the brain to the intestines	Reduces alcohol intake	Nevo and Hamon 1995
Cholecystokinin	Conveys excitatory signals from the brain to the intestines	Reduces alcohol intake	Nevo and Hamon 1995
Dopamine	Conveys excitatory signals from one neuron to another	Acute alcohol ingestion facilitates dopamine’s excitatory effect	Nevo and Hamon 1995
		Acute alcohol withdrawal reduces dopamine’s excitatory effect	Nevo and Hamon 1995
Glutamate	Conveys excitatory signals from one neuron to another	Acute alcohol ingestion reduces glutamate’s excitatory effect	Nevo and Hamon 1995
Monoamine oxidase (MAO)	Catalyzes the breakdown of dopamine and serotonin	Unknown	Nevo and Hamon 1995
Norepinephrine	Conveys excitatory signals from one neuron to another	Acute alcohol ingestion facilitates NE’s excitatory effect	Nevo and Hamon 1995
Peptides	Convey excitatory signals from one neuron to another	Lead to a global reduction in the production of peptides	Madeira and Paula-Barbosa 1999
Serotonin	Conveys excitatory signals from one neuron to another	Acute alcohol ingestion facilitates serotonin’s excitatory effect	Yoshimoto et al. 2000
		Chronic alcohol ingestion reduces serotonin’s excitatory effect	Berggren et al. 2002

* These actions represent the primary effects of the various neurotransmitters; however, depending on the brain region and cell type studied, each transmitter also may have other effects.

Although autopsy studies can provide valuable information, imaging studies in living humans beings often are preferable, particularly when investigating the progression of alcohol-related brain damage or when determining alcohol’s effects on brain function. Structural imaging techniques such as CT and MRI (Wong and Brašić 2001) have confirmed the findings of brain shrinkage and reduced the number of brain cells in living subjects with Wernicke’s encephalopathy and other disorders associated with alcoholism (Viola et al. 2001). Additionally, DTI studies of alcoholics suggest the presence of abnormalities in the white matter of the brain, which consists of the extensions (i.e., axons) of neurons (Pfefferbaum and Sullivan 2002; Sullivan and Pfefferbaum 2003). Brain shrinkage and other abnormalities primarily affect the frontal lobes (Moselhy et al. 2001), although shrinkage also occurs in other brain regions in people with chronic excessive alcohol consumption.

Imaging analyses that have identified structural brain changes are complemented by functional imaging methods such as PET, which reveal changes in blood flow and other metabolic activities associated with specific sensory, motor, or cognitive functions and are impaired in people with alcohol dependence. (It is important to note, however, that neuropsychological changes may not necessarily correlate with the metabolic changes seen on PET scans of alcoholics.)

When conducting PET analyses, researchers often perform two scans on each participant to study metabolic changes throughout the brain that may be associated with particular activities. The first scan typically is performed when the patient is in a resting state to determine the basal metabolism of the stable brain. The second scan is performed during the activated condition—that is, after exposure to a psychological or pharmacological stimulus. For example, psychological activation can be accomplished by engaging the person in an activity such as viewing a videotape or performing a mental task. Alternatively, pharmacological activation may consist of administering a pharmacological agent such as an amphetamine to simulate the maximal release of dopamine in physiological excitation or stress (Wong and Brašić 2001). The findings of such analyses are summarized in the following sections.

## Effects of Chronic Alcohol Consumption on Neurotransmitters

### Overview of Neuronal Communication

To understand how chronic excessive alcohol use associated with alcohol dependence affects brain function, it is important to understand how neurons communicate with each other through electrical and chemical signals. Nerve signals are transmitted from one region of the brain to another region of the brain or to the rest of the body through serial communication between two or more neurons located next to each other. When a neuron is activated, an electrical signal is generated (usually near the neuron’s body), which travels along the membrane surrounding the cell body and the long extension protruding from it (i.e., the axon). When the signal reaches the end of the axon, it triggers the release of neurotransmitters from the cell. These neurotransmitters travel across the narrow space separating one neuron from another (i.e., the synaptic cleft). On the signal-receiving neuron, the neurotransmitter molecules then interact with receptors, and this interaction either promotes or prevents the generation of new electrical signals in that neuron, depending on the neurotransmitter. Neurotransmitters that promote the generation of a new nerve signal are called excitatory neurotransmitters; those that prevent the generation of a new nerve signal are called inhibitory neurotransmitters. Many neurotransmitters can have both excitatory and inhibitory effects, depending on which brain region is studied and which receptors are present on the signal-receiving neurons. Neurotransmitters that often have excitatory effects include dopamine, glutamate, and serotonin; neurotransmitters that primarily have inhibitory effects are GABA and glycine. (For a list of excitatory and inhibitory neurotransmitters that may play a role in alcohol’s actions, see the table, p. 164).

Alcohol’s effects on the brain are mediated by numerous neurotransmitters and their highly complex interactions. In general, the pleasurable psychological experiences associated with alcohol consumption appear to be mediated by dopamine, noradrenaline, and the endogenous opioids and their receptors (Basavarajappa and Hungund 2002). Other neurotransmitters commonly affected by alcohol are glutamate and GABA.

### Alcohol’s Effects on Inhibitory Neurotransmitters

Alcohol is thought to influence two inhibitory neurotransmitters—GABA (Korpi et al. 2002; Nevo and Hamon 1995) and glycine. Alcohol appears to enhance the inhibitory actions of GABA (Nevo and Hamon 1995), which may contribute to both the acute and the chronic effects of alcohol and to the phenomena of alcohol dependence, tolerance, and withdrawal (Nevo and Hamon 1995). Chronic alcohol consumption leads to a decline in the number of GABA receptors in the brain and reduces GABA’s ability to bind to its receptors, thereby allowing the body to compensate for the alcohol-induced enhancement of GABA’s actions. These effects are a part of the changes in brain function that lead to tolerance and dependence on alcohol (Nevo and Hamon 1995). When alcohol is withheld, however, and its stimulating effect on GABA is eliminated, the body suddenly has too few GABA receptors to balance the actions of the excitatory neurotransmitters. As a result, the brain experiences an excess of excitatory nerve signals, a phenomenon known as rebound hyperexcitability. This hyperexcitability may contribute to the physical and psychological manifestations of alcohol withdrawal (Nevo and Hamon 1995).

Alcohol’s effects on the inhibitory neurotransmitter glycine are controversial, however. Studies have found that both acute and chronic alcohol consumption exerted only minimal effects on the role of glycine in the nervous system (Nevo and Hamon 1995).

### Alcohol’s Effects on Excitatory Neurotransmitters

Alcohol consumption appears to influence the transmission of signals mediated by many excitatory neurotransmitters, most prominently glutamate, dopamine, and serotonin (Nevo and Hamon 1995).

#### Glutamate

Glutamate exerts its effects by interacting with several types of receptors, including one called the *N*-methyl-d-aspartate (NMDA) receptor. Alcohol acts on these NMDA receptors, inhibiting their functions and thereby diminishing glutamate-mediated neurotransmission. NMDA receptors may play a role in memory formation; prenatal, acute, or chronic alcohol exposure may hinder the person’s ability to learn and to retain new information (Nevo and Hamon 1995).

#### Dopamine

**Figure 3:**
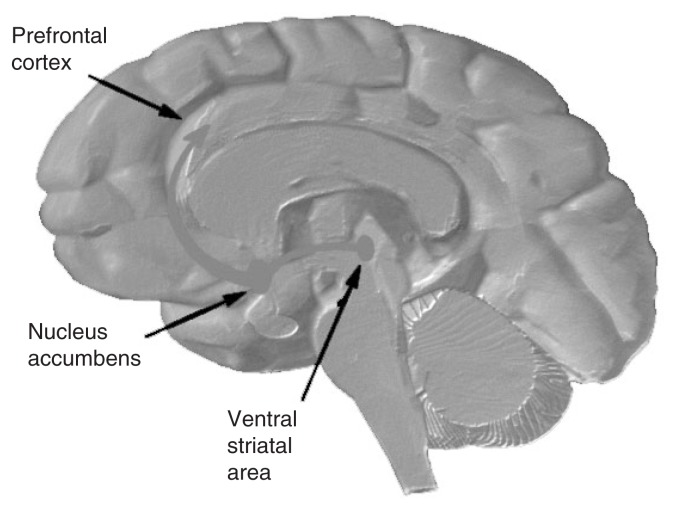
A diagram of the right half of the brain, as viewed from the inside cut surface. The left side of the figure is the frontal or anterior end of the brain; the right side of the figure is the occipital or posterior end of the brain; the top of the figure is the superior or top side of the brain; and the bottom of the figure is the inferior or lower side of the brain. SOURCE: National Institute on Drug Abuse (NIDA) 2003. [Online at http://www.drugabuse.gov/pubs/teaching/Teaching3/largegifs/slide-4.gif.]

In contrast to its dampening effects on the activity of the glutamate system, acute alcohol ingestion enhances the excitatory effect of dopamine (Nevo and Hamon 1995). Correspondingly, acute withdrawal from alcohol reduces dopamine’s excitatory effect. PET studies have confirmed that dopamine and its actions in the brain are involved in the subjective experience of reward (Koob and Weiss 1992; Oswald et al. 2003). Anatomically, the reward system is located deep in the brain in a region called the ventral striatal area, with nerve fibers projecting to an area known as the nucleus accumbens and subsequently to higher regions of the brain (see figure 3). This also is called the mesolimbic dopamine system. Alcohol and other drugs (AODs), as well as food or sex, can trigger the release of dopamine in this reward system and reinforce the subjective pleasurable experiences therefore associated with alcohol or the other stimuli and are a component of the reward process. PET studies have allowed researchers to directly investigate the role of dopamine and the reward system in alcohol consumption in humans (Oswald et al. 2003).

When alcohol induces the release of dopamine in the nucleus accumbens, nerve signals are sent to the cortex, where they are registered as “experience” and memories of the rewarding effects of alcohol, such as its taste or the feelings of relaxation after drinking. Once registered, these memories can stimulate further alcohol intake, completing the reward system. Because memories of the rewarding effects of alcohol also include the environment in which drinking occurred, even sights or smells related to that environment can subsequently trigger the reward system. Indeed, several studies have suggested that alcoholics are predisposed to relapse and that environmental stimuli related to alcohol can trigger the impulse to drink (Flannery et al. 2001). Animal studies have confirmed that the nucleus accumbens is probably involved in the rewarding aspects of alcohol consumption and also may mediate the stimulatory effects of environmental cues associated with past drinking (Katner and Weiss 1999). Another study using single photon emission computed tomography (SPECT) found that alcoholics ingesting a sip of alcohol during brain imaging showed enhanced neuronal activity in a certain region of the ventral striatal area (i.e., a part of the basal ganglia) that correlated highly with their increase in craving (Modell et al. 1990). Because alcohol consumption increases dopamine release preferentially in the ventral striatal area, these findings support the view that dopamine activation is a common property of AODs and contributes to their reinforcing effects.

Recent studies have suggested a link between stress and altered activity of the mesolimbic dopamine system. Stressful situations result in the increased release of hormones called glucocorticoids, most prominently cortisol. Studies have found that glucocorticoids can increase mesolimbic dopamine release (Piazza and Le Moal 1996; Biron et al. 1992; Fahlke et al. 1994; Piazza et al. 1994). It has been suggested that the stress-induced increase in dopamine release may make the person more sensitive to the rewarding effects of AODs, which may represent one of the pathways leading to abuse of those drugs (Deroche et al. 1995; Piazza et al. 1990; Kalivas and Stewart 1991). Recently, researchers have utilized PET to study the relationship between cortisol release and amphetamine-induced dopamine release (Maini et al. 2003). These preliminary studies, which suggest a high correlation between cortisol release and dopamine release, may open the way for future studies of these relationships in alcoholics and their relatives. Other studies have found that actively drinking alcoholics appear to have an abnormal hormonal response to stress, which also may be present in the offspring of alcoholics who are not yet heavy drinkers (Wand et al. 1998, McCaul et al. 2000).

#### Serotonin

Serotonin, another excitatory neurotransmitter involved in the brain’s reward system, appears to play an important role in alcohol abuse. As with dopamine, animal studies have demonstrated that acute alcohol administration resulted in enhanced serotonin release (Yoshimoto et al. 2000), and withdrawal from alcohol was associated with reduced serotonin release (De Witte et al. 2003). Moreover, studies have found that alcoholics with years of excessive alcohol consumption appeared to exhibit impaired serotonin and dopamine activity (Berggren et al. 2002).^5^ Finally, studies using SPECT found a genetic defect in the gene encoding a serotonin transporter in some people who were particularly sensitive to the toxic effects of chronic excessive alcohol consumption on the brain (Heinz et al. 2000). The serotonin transporter is a protein located in serotonin-producing neurons that removes serotonin from the space between neurons to stop serotonin’s effect on the signal-receiving neuron. Thus, people with abnormal serotonin transporter function may be particularly susceptible to the reduced excitatory effect of serotonin caused by heavy alcohol consumption. The reduced effect of serotonin, in turn, probably leads to reduced effects of dopamine. Thus, alcoholics with abnormal serotonin transporter function are likely to need greater amounts of alcohol to attain the pleasurable feelings associated with alcohol consumption (Heinz et al. 2000).

**Glossary of Terms**

Antagonist: A chemical compound whose physiological effect is the opposite of the effect created by the original molecule. For example, a *dopamine* antagonist has the opposite physiological effects from those of dopamine.
Atom: The chemical unit of matter.
Axon: The long nerve fiber extending from the body of the *neuron*.
Computerized tomography (CT): A computer-assisted technique that generates visual cross-sectional images by exposing a subject to an x-ray beam that rotates around the subject and then recording those beams that pass through the body.
Cyclotron: A machine that creates radioactive compounds.
Diffusion tensor imaging (DTI): A technique for examining the integrity of the microstructures of tissues, including *axons*.
Dopamine: An excitatory *neurotransmitter* that plays a role in the reward system in the brain and possibly also in the reinforcing properties of alcohol.
Electron: A negatively charged particle within an *atom*.
Emission: The release of radioactivity from a radioactive source.
Excitatory neurotransmitter: A *neurotransmitter* that promotes the generation of a new nerve signal in the signal-receiving *neuron*.
[^18^F]fluorodeoxyglucose (FDG): A *radiotracer* used to assess utilization of the sugar glucose by the body and the brain.
Functional imaging: Techniques for obtaining images that represent physiological and metabolic processes performed by the organs of the body.
Gamma-aminobutyric acid (GABA): An inhibitory *neurotransmitter* whose actions are influenced by alcohol; may play a role in alcohol withdrawal.
Glutamate: An excitatory *neurotransmitter.*
Gray matter: Portions of the nervous system with a gray color; the gray matter primarily contains the bodies of nerve cells.
Half-life: The time during which the radioactivity contained in a compound decreases by one-half.
Inhibitory neurotransmitter: A *neurotransmitter* that prevents the generation of a new nerve signal in the signal-receiving *neuron*.
Magnetic resonance imaging (MRI): A computer-assisted technique for creating cross-sectional images by exposing a subject to radio waves in the presence of a powerful magnetic field and measuring signals emitted by certain *atoms* in the affected area in response to this treatment.
Metabolism: The sum of all biochemical processes in a living organism; also the production and breakdown of a given compound.
Myelin: Protective covering that facilitates cell-to-cell communication.
Neuroimaging: Visual representation of the nervous system.
Neuron: A nerve cell.
Neurotransmitter: A chemical (e.g., *dopamine*, *GABA*) that conveys a signal from one *neuron* to another.
Nucleus: The positively charged, dense center of an *atom* that contains most of the weight of the atom; contains *positrons.*
Photon: A particle of light.
Positron: A positively charged particle located in the nucleus of an *atom;* has the same weight as an *electron*.
Positron emission tomography (PET): A computer-assisted technique for generating cross-sectional images of a subject by measuring the radioactivity released by *radiotracers* within the subject’s body.
Radiotracer: A radioactive compound administered to a subject in order to localize specific chemicals in the body.
Receptor: A complex of one or more proteins on the surface of a cell that binds to a specific chemical (e.g., a *neurotransmitter*).
Regional cerebral blood flow (rCBF): The flow of blood through a part of the brain.
Resolution: The smallest detectable distance between two points.
Single photon emission computerized tomography (SPECT): A computer-assisted technique for generating cross-sectional images of a subject; combines the use of *radiotracers* with the computer technology used in computed *tomography*.
Structural imaging: An imaging technique for analyzing the anatomic relationships of organs, cells, and subcellular structures.
Superior frontal cortex: The layer of nerve cells covering the upper surface of the front of the brain.
Tomography: The visual presentation of cross-sectional slices through an object.
White matter: Portions of the nervous system with a whitish color; consists primarily of the *axons* of nerve cells that are wrapped by the whitish protein *myelin*.

One goal of research on serotonin and other neurotransmitters in alcoholism is to identify distinct biological subtypes of alcoholism and biological markers for them, which may then help to develop more targeted treatment approaches. For example, if one biological subtype of alcoholism was characterized by defective serotonin transporter function, brain scans for the presence of the serotonin transporter could serve as a tool to obtain a biological marker for this alcoholism subtype. Similarly, repeated scans after the administration of a potential treatment for the serotonin transporter deficiency could help identify the effect of that treatment. Future studies of the effects of chronic alcohol consumption on the serotonin system may clarify the role of serotonin and dopamine in alcoholism subtypes. Neuroimaging techniques may help to identify the specific chemicals, such as dopamine and serotonin, that are deficient in particular biological subtypes of alcoholism, and to monitor the effects of potential therapies targeted for the specific deficiency of the biological subtype (Wong et al. 2002).

#### Other Neurotransmitters

In addition to glutamate, dopamine, and serotonin, alcohol also acts on various other excitatory neurotransmitters conveying signals within the brain as well as to other organs, as follows (also see the table, p. 164):

- Acute administration of alcohol increases the excitatory effects of the neurotransmitter norepinephrine (Nevo and Hamon 1995).
- Acetylcholine is an excitatory neurotransmitter that among other functions plays a role in memory. Chronic consumption of alcohol reduces the number of neurons containing acetylcholine (Nevo and Hamon 1995). This reduction may be associated with the memory deficits commonly associated with heavy chronic alcohol consumption.
- Bombesin and cholecystokinin are compounds produced in the brain that stimulate the functioning of the intestines. Alcohol does not appear to influence the actions of these compounds, but both bombesin and cholecystokinin reduce the intake of alcohol (Nevo and Hamon 1995).

### Alcohol’s Effects on Endogenous Opioids

Endogenous opioids are molecules produced in the body that resemble opium; they apparently act like excitatory neurotransmitters to stimulate neurons. It is hypothesized that endogenous opioids reinforce the effects of alcohol and play a role in the pleasurable effects of both acute and chronic alcohol consumption, but their specific part in alcohol abuse and dependence remains to be clarified (Nevo and Hamon 1995). What is known is that alcohol influences one of the opioid receptors—the mu receptor—in the brain. For example, chronic heavy drinkers have alterations of mu receptors in neurons both in the outer layer of the brain and in structures deep in the center of the brain (Bencherif et al. 2004)**.** In addition, studies have found that a medication called naltrexone that inhibits opiate receptors in the brain is an effective treatment for alcoholism (Romach et al. 2002; Terenius 1996), particularly for people with a family history of alcoholism or with a strong craving for alcohol (Monterosso et al. 2001). Other studies have found that alcoholics carrying a specific variant of the mu receptor have a lower relapse rate after treatment with naltrexone than do those carrying other receptor variants (Oslin et al. 2003). These findings suggest that alcoholics with a particular genetic makeup are particularly likely to benefit from treatment with naltrexone. Because PET technology offers promise as a tool for determining the density and the distribution of mu opiate receptors in the brain, this technique may help identify alcoholics who could benefit from interventions such as naltrexone, which affect these receptors. Thus, PET studies to identify mu opiate receptors in the brain may be a tool for identifying a distinct biological subtype of alcoholism; and PET findings could serve as a biological marker of mu opiate receptor dysfunction in the brain (Wong et al. 2002).

## PET Studies of Brain Glucose Metabolism and Blood Flow

### Glucose Metabolism

To function properly, the brain needs a continuous supply of the sugar glucose, whose breakdown provides most of the energy the cells need for their diverse functions. Brain regions that are more active, including the cells of rapidly growing tumors, require more glucose. Similarly, lower-than-normal glucose metabolism suggests reduced brain activity indicative of neurological or cognitive problems. PET studies can help researchers identify brain regions that are active at any given time by administering radioactively labeled glucose (i.e., [^18^F]fluorodeoxyglucose [FDG]) and measuring its distribution in the brain. Brain glucose metabolism detectable with PET occurs mainly in the gray matter—the brain regions where the bodies of neurons are located. The amount (or volume) of gray matter in the brain, however, can vary substantially among subjects. For example, chronic alcoholics frequently have smaller gray-matter volumes than nonalcoholics (Sullivan 2000). Therefore, data regarding glucose metabolism must be expressed in terms of the gray-matter volume of a specific region, which can be determined by structural imaging techniques such as MRI.

PET studies have shown that glucose metabolism in alcoholics is decreased in the entire brain (Volkow et al. 1992), with the most marked reductions in the frontal lobes and cerebellum. However, an assessment of the effects that reduced glucose metabolism may have on brain functioning in people with alcohol dependence is complicated by the alcohol-induced damage to other organs (e.g., the liver, stomach, or other vital organs) often found in those people. For example, people with liver cirrhosis resulting from chronic alcohol consumption exhibit decreased glucose utilization by gray matter in the frontal and temporal lobes as well as the basal ganglia (Kato et al. 2000). Thus, neurological and cognitive problems of alcoholics may not only be a consequence of reduced glucose metabolism but may reflect the effects of alcohol-induced liver, kidney, and heart dysfunction on the brain. Furthermore, glucose may play a different role in brain metabolism in alcoholics with clear neurological or cognitive problems than in healthy people. Further research is needed to clarify glucose metabolism in alcoholics with neurological and cognitive problems.

### Regional Blood Flow

Glucose is brought to the brain via the bloodstream; accordingly, the rates of regional cerebral blood flow (rCBF) within various areas of the brain are regulated depending on the changing demands of these areas. This variability in blood flow depending on regional brain activity is the basis for using PET to measure rCBF. To detect changes in rCBF, investigators inject a radiotracer (typically radioactively labeled water, [^15^O]H_2_O) into the bloodstream and measure its deposition in the brain tissue, which is determined by the regional distribution of blood flow. ^15^O has a short half-life of 2 minutes and therefore can be injected repeatedly while the subjects perform various motor, sensory, or cognitive tasks under different conditions. Assessing the differences in blood flow between tasks enables investigators to identify the brain regions involved in each specific task. This approach can also be used to track the effects of acute alcohol ingestion on regional blood flow over a period of time (Sullivan 2000).

## Correlating Structural and Physiological Changes With Alcohol-Related Behaviors

Once PET and other studies have identified changes in brain structure and functioning of alcoholics, investigators must correlate these changes to alcohol-related behaviors in those patients. For example, studies have linked both shrinkage of the cerebellum and decreased blood flow in this region, as determined by imaging studies, to impaired balance and gait, which may cause falls, particularly in older alcoholics (Volkow et al. 1988). Falls can result in head injuries and further deterioration in brain function. Other functional imaging studies have shown that decreases in blood flow and metabolism in the frontal lobes precede shrinkage of that brain region and major cognitive abnormalities (Johnson-Greene et al. 1997).

Imaging studies also have demonstrated that cognitive functions and motor coordination may improve partially within 3 to 4 weeks of abstinence and that these improvements are accompanied by a partial reversal of brain shrinkage (Johnson-Greene et al. 1997). Frontal lobe blood flow also increases with abstinence, returning to normal levels within 4 years, whereas a relapse to drinking leads to renewed brain shrinkage and blood flow reductions (Johnson-Greene et al. 1997).

Finally, PET studies have helped researchers assess risk factors for alcoholism. In nonalcoholics, certain sedatives (i.e., benzodiazepines) produce a temporary impairment in coordination and cognition and a decrease in brain glucose metabolism similar to the effects of alcohol consumption. In alcoholics, however, some regions in the frontal lobe respond to benzodiazepines less strongly than they do in nonalcoholics (Gilman et al. 1996). These results suggest that alcoholics may have a diminished capacity to dampen excessive neuronal activity and therefore may be less able to inhibit behavior.

## Methodological Considerations for PET Studies in Alcoholics

### Developing Models for Interpreting Findings of PET Analyses

The data obtained in alcoholics with functional imaging techniques such as PET typically must undergo a set of processing steps to yield information that is useful to researchers. For example, researchers must develop mathematical representations (i.e., kinetic models) of physiological processes such as the metabolism of neurotransmitters or their receptors. With these models, investigators can develop a mathematical equation describing the tissue response curve expected in the measurements. The tissue-response curve plots the radioactivity of specific parts of the brain before, during, and after the injection of the radiotracer. Thus, the tissue-response curve correlates with the amount of the chemical identified by the radiotracer in the regions of interest. By performing the scans on groups of people with and without alcoholism, the increases and decreases of chemicals in the brains of alcoholics can be identified. By identifying those variables in the model that give the best agreement between the expected and measured values, one can quantify the physiological process.

To develop appropriate models and provide a basis for interpreting the measured behavior of the tracer, all available qualitative information about the physiology and biochemistry of the tracer is collected. For example, it is important to know how fast and to what extent the tracer is transported from the site of the injection in the bloodstream to the tissue being analyzed (e.g., a specific brain region). The basic steps of this transport are as follows:

- The tracer is transported by the blood to the small blood vessels (i.e., capillaries) in the brain.
- The tracer moves across the capillary wall into the fluid-filled spaces between the brain cells.
- The tracer crosses the membrane surrounding the cells or binds to neurotransmitter receptors in the synaptic clefts between neurons.
- If it enters the cells, the tracer participates in various biochemical reactions.

PET can follow the progress of the tracer by measuring the amounts of radioactivity in different areas of the brain as well as the tracer concentrations in the blood. To interpret the data obtained in this way, investigators can use a variety of mathematical or statistical modeling methods (e.g., the compartment model, graphical model, and tissue input graphical model approaches). In many cases, researchers attempt to simplify their models by making assumptions about the processes involved in the model (e.g., about how easily the tracer can cross the capillary walls). To make sure these assumptions are reasonable or correct, however, the simplified model must first be validated. To this end, the investigators must make sure that the model yields reasonable values for the variables tested and that it can distinguish the disease state (e.g., conditions in an alcoholic) from the healthy state (e.g., conditions in a nonalcoholic).

### Correcting for Partial Volume Errors

Compared with structural imaging techniques (e.g., MRI), PET images are blurred because of the limited resolution of the PET scanners (i.e., their limited ability to distinguish closely spaced regions of small dimensions). This limited resolution has two potential consequences:

- An apparent loss (or “spill-out”) of radioactivity signals from a small region of interest into the adjacent tissues owing to the size of the small brain region compared with the spatial resolution of the scanner
- A “spill-in” of radioactive signals into the region of interest from adjacent brain areas with different radioactive tracer concentrations.

These effects, which are known as “partial volume errors,” are more pronounced in alcoholics with alcohol-related brain shrinkage, where loss of signal because of partial volume errors could be confounded with an actual loss of tissue function (Rousset et al. 1998).

Several methods are available to correct for this problem. The most common approach is to perform both an MRI scan and a PET scan of a patient’s brain and then to combine the images using several available methods (see figure 1). Computer simulations then are used to mimic the effect of limited spatial resolution to characterize the partial volume effects for each brain region (Rousset et al. 1998). With this information, investigators then can apply correction factors to obtain more accurate estimates of the actual regional activity (e.g., regional blood flow or glucose metabolism).

## The Future of PET Studies in Alcoholism

Although researchers have been employing PET and other functional imaging techniques in the analysis of alcohol-induced changes in brain functioning, the full potential of these approaches has not yet been realized. For example, it might be useful to correlate functional imaging data with information on demographic traits of the subjects (Brašić 2003) as well as with behavioral measures, including questionnaires addressing psychological traits and the desire for alcohol. Demographic and psychological traits may identify biological subtypes of alcoholism detected by PET. Questionnaires to identify behavioral data could function as biological markers for the presence of distinct biological variants of alcoholism and could help identify the effects of potential therapeutic interventions targeted to the distinct variant (Wong et al. 2002). Other possible applications of PET include the following:

- Studies in humans and animals to characterize neurochemical processes associated with alcohol reinforcement and/or craving, such as the production, release, and transport of neurotransmitters or changes in receptor concentrations
- Identification of neural circuits that play a role in the cognitive deficits associated with acute alcohol intake as well as elucidation of the pathways through which functional deficits in specific neural circuits and the resulting cognitive deficits may contribute to excessive alcohol intake
- Analyses of neurobiological markers of vulnerability to alcohol abuse
- Combination with structural imaging techniques (e.g., MRI or CT) to obtain a fused image automatically (see figure 1)
- Development of new pharmaceutical agents to prevent and treat alcoholism (Wong et al. 2002).

## Summary

PET allows researchers to visualize in living human beings the damage to the brain that results from chronic excessive alcohol consumption. This technology has been used to analyze alcohol’s effects on various neurotransmitter systems as well as on glucose metabolism and regional blood flow in the brain. Such analyses have detected deficits in alcoholics, particularly in the frontal lobes, which control numerous cognitive functions, and in the cerebellum, which controls voluntary movements. In addition, PET is a promising tool for monitoring the effects of alcoholism treatment and abstinence on damaged portions of the brain. Finally, PET may be able to help researchers develop new medications targeted at correcting the chemical deficits found in the brains of people with alcohol dependence and alcohol abuse.

## Footnotes

1 For a definition of this and other technical terms used in this article, see the glossary, pp. 170–171.

2 The MRI–PET procedure described here is time consuming and technically demanding and can therefore be used only in a few specialized research settings, but is not widely available for clinical purposes.

3 If a large amount of radiotracer was administered, the sudden excess of the compound under investigation could alter the rate or location of the biological processes in which that compound is involved. In general, the dose of a radiotracer for a routine PET scan is roughly 1,000 times (or three orders of magnitude) lower than the dose required to produce a pharmacological effect.

4 The blood–brain barrier is a physiological property of the blood vessels in the brain that prevents many substances from entering the brain, thereby protecting the brain from potentially harmful molecules.
